# A systematic review of the impact of intensive care admissions on post discharge cognition in children

**DOI:** 10.1007/s00431-021-04145-5

**Published:** 2021-06-11

**Authors:** Ana Sánchez-Moreno Royer, Jamiu O. Busari

**Affiliations:** 1 Faculty of Health, Medicine and Life Sciences, Maastricht University, Universiteitssingel 40, 6229ER Maastricht, Netherlands; 2 Faculty of Health, Medicine and Life Sciences, Educational Development Research Department, Maastricht University, Universiteitssingel 40, 6229ER Maastricht, Netherlands; 3 Department of Pediatrics and HOH Academy, Horacio Oduber Hospital, Dr. HE Oduber Boulevard #1, Oranjestad, Aruba

**Keywords:** Cognition, Cognitive development, PICU (hospitalization), NICU (hospitalization), Post-discharge, Systematic development

## Abstract

Understanding how hospitalization affects cognitive development is crucial to safeguard children’s cognition; however, there is little research evaluating the associations between NICU or PICU hospitalization and survivors’ cognition. The objective of this study is to identify and characterize the associations between a neonatal or pediatric ICU hospitalization and the short- and long-term cognition of survivors. The databases Cochrane Library, Medline, EBSCO, Embase, and Google Scholar and the journals JAMA Pediatrics, Journal of Pediatrics, Pediatrics, Archives of Disease in Childhood, Academic Pediatrics, Pediatric Critical Care Medicine and Child Development were searched until April 2021. Retrieved article references were analyzed. Included articles investigated cognition as an outcome of ICU hospitalization in non-preterm neonatal or pediatric patients. Case studies and studies analyzing diagnosis or treatment interventions were excluded. Four prospective cohort or case-control studies and two retrospective cohort studies were included, totaling 2172 neonatal and 42368 pediatric patients. Quality assessment using the BMJ Criteria and Cochrane Collaboration’s Risk-of-Bias tool displayed good results. Significant negative associations were found between neonatal cognition and length-of-ICU-stay at 9- (*p*<0.001) and 24 months (*p*<0.01), and between pediatric cognition and length-of-ICU-stay at discharge (*p*<0.001). Additional weeks on the neonatal ICU increased odds of impairment at 9- (OR 1.08, 95%CI 1.034–1.112) and 24 months (OR 1.11, 95%CI 1.065–1.165).

*Conclusion*: There is a significant negative correlation between NICU and PICU hospitalization and the short- and long-term cognitive status. Future research must identify patient- and hospital-related risk factors and develop management strategies.

**Table:** 

**What is Known:** *• Cognitive development relies on the presence of stimulating factors and absence of risk factors, and is hypothesized to be directly and indirectly affected by hospitalization in the short and long term.* *• No research examines the relation between survivor cognition post-discharge of a general pediatric hospitalization, and scarcely more of a neonatal or pediatric intensive care hospitalization.*	
**What is New:** *• NICU and PICU hospitalization is independent risk factors for survivor impaired cognition in the short and in the long term with a dose-response effect. High risk patients for cognitive impairment should be identified and appropriately followed-up.* *• Patients with an ICU hospitalization of over 2.5 days and two or more of the following factors should be considered high risk: increased mortality risk, invasive interventions, neurological or oncological diagnosis, postnatal complications or decreased maternal mental health status.*

**What is Known:**

*• Cognitive development relies on the presence of stimulating factors and absence of risk factors, and is hypothesized to be directly and indirectly affected by hospitalization in the short and long term.*

*• No research examines the relation between survivor cognition post-discharge of a general pediatric hospitalization, and scarcely more of a neonatal or pediatric intensive care hospitalization.*

**What is New:**

*• NICU and PICU hospitalization is independent risk factors for survivor impaired cognition in the short and in the long term with a dose-response effect. High risk patients for cognitive impairment should be identified and appropriately followed-up.*

*• Patients with an ICU hospitalization of over 2.5 days and two or more of the following factors should be considered high risk: increased mortality risk, invasive interventions, neurological or oncological diagnosis, postnatal complications or decreased maternal mental health status.*

## Introduction

Rapid improvements in pediatric critical care medicine have led to more pediatric patients surviving intensive care unit (ICU) hospitalizations and their successful rehabilitation into society. While much has been invested in what happens up to and during hospitalization—disease diagnosis, treatment and prognosis—there remains a poor understanding of the consequences of ICU hospitalization in and of itself on the well-being of children post-discharge [1] [2], particularly on their cognitive well-being [3] [4]. An association between ICU hospitalization and cognition would have important clinical consequences on patient management and survivor’s quality of life. It is therefore necessary to improve our understanding in order to protect and stimulate children’s cognitive status and development.

It is hypothesized that hospitalization, in particular an ICU hospitalization, may have an association with cognitive delays and deficits through various mechanisms. ICU hospitalization is a major life event for both children and families [2, 5], which can constitute early adversity and result in impairment in various fields [4–6]. During ICU hospitalization, risk factors for cognitive impairment are multiplied [4, 6–8], while positive cognitive stimuli are reduced [9–11]. Relevant stressors include risk factors such as exposure to strangers, medical environments and psychological stress as well as removal of protective factors such as separation from the family and familiar environments [4, 6, 8]. Important cognitive stimuli vary based on age, however main eliminated stimuli include exposure to sensory stimuli, exercise, playing and schooling [9–13].

Neonatal ICU (NICU) and pediatric ICU (PICU) survivors have been found to suffer from common psychiatric disorders, including depression, anxiety and post-ICU syndromes [5, 6, 13, 14], and from new or increasing impairments in various cognitive functions [6–8]. Major identified risk factors which account for these impairments are both the critical illness itself in addition to elements related to hospitalization in and of itself. Surgical interventions, mechanical ventilation, sedation, and pain medication are linked to psychological stress, and possible subsequent cognitive difficulties [5, 6, 15]. Patients describe uniformed personnel, fear of the unknown and of pain, and hospital design to be sources of stress [16, 17]. Deprived environments, such as hospitals or institutions, have also been linked to neuropsychiatric morbidities and impairments in memory, executive function and social interactions [8–10, 18–20].

While there is no consensus regarding the repercussions of PICU hospitalization on cognition, the theoretical background and preliminary findings strongly suggest that hospital admission constitutes a period of vulnerability during which cognitive development is both directly and indirectly affected. Although hospitals have adapted to minimize the physical and psychiatric consequences of critical illness and hospitalization, the lack of research regarding cognitive consequences has limited their ability to prevent cognitive impairments. A review of the literature is necessary to understand the prevailing knowledge on the association between ICU hospitalization and cognitive development and to establish future steps for research in this area. This will assist in promoting complete recovery, with a focus on preventing cognitive impairment and stimulating cognition, in order to guarantee future quality of life.

The focus of this review lies in ICU hospitalization for two main reasons. First, compared to a general hospitalization, an ICU admission is more severe and has a greater influence post-discharge on survivors [6–8]. As examining the hypothesized association is complicated, it is hoped that choosing to focus on an ICU hospitalization will facilitate establishing and analyzing this possible association. Secondly, a preliminary literature search revealed that no research had been performed studying the association between general hospitalization and cognitive outcomes. From a practical point of view, it was therefore impossible to focus on a general hospitalization, or any other kind of hospitalization, other than an ICU hospitalization. It is hoped that by performing a systematic review focused on ICU hospitalization, it will be possible to extrapolate results into future research focused on general hospitalization and improve treatment and management of NICU and PICU survivors.

The objective of this systematic review is to compile and comprehend current knowledge concerning the association between ICU hospitalization and neonatal and pediatric survivor cognitive status and development. This includes determining relevant patient and hospital-related risk and protective factors for this association. To achieve these objectives, all articles examining the connection between a NICU and PICU hospitalization event on survivors’ cognition in the short- and long-term were reviewed.

## Methods

The Medline search strategy was adopted with a broad approach to ensure all possible articles were included. This broad strategy was employed due to the dearth of research regarding the association between ICU hospitalization and cognition in survivors. The search was conducted until April 2021 with language limited to English, Dutch, Spanish, French, and Portuguese and no publication date limitations.

To ensure completeness, both the databases Cochrane Library, PubMed, Google Scholar, Embase and EBSCO and the journals JAMA Pediatrics, Journal of Pediatrics, Archives of Disease in Childhood, Pediatrics, Academic Pediatrics, Pediatric Critical Care, and Child Development were searched. The databases and journals cover the highest impact, best reputed pediatric resources. Reference searches of retrieved articles were also assessed for inclusion. Combinations of the following keywords were used for the search:

- Population: pediatric, neonatal, infant(s), child(ren)
- Variable: hospitaliz(s)ation, hospital admission
- Outcome: cognitive, cognition, development, learning, language, memory, delay, disorder(s), growth/developmental milestone(s)
- Other: consequence, influence, impact

Articles were included if these criteria were met: NICU or PICU hospitalization as the main variable, cognitive status or development as the main outcome, and neonatal or pediatric patients aged between 0 and 21 years as participants. This wide age range was chosen because cognitive development continues past legal adult age into the early twenties. Studies analyzing specific diagnosis or treatment interventions, or including preterm patients were excluded to avoid additional comorbidities which could constitute confounders. Case studies were also excluded.

Article quality was assessed using the BMJ guidelines and Cochrane Collaboration’s Risk-of-Bias tool on these elements: selection process, outcome measurement, follow-up, bias risk (selection, information, observer, publication bias), confounding variables, indirectness, and inconsistency. Certainty was increased for large magnitude of effect, dose-response gradients, and for highest quality evidence. In a first round, data extraction, grading and review was performed independently by both authors. In a second round, consensus was reached through discussion and secondary assessment to establish data extraction, final grade and review conclusions. Data extraction was performed manually based on research objectives. Levels of evidence were additionally evaluated using the Oxford Centre of Evidence Based Medicine guidelines [21], and the JAMA Pediatrics Quality Rating tools [22].

There was no missing data or missing articles as all available articles and data were included. Due to the clinical differences and the heterogeneity in collected data concerning cognitive outcomes between the included articles, a meta-analysis was not performed to prevent drawing inappropriate conclusions.

## Results

### Literature search, article selection, and assessment

**Fig. 1:**
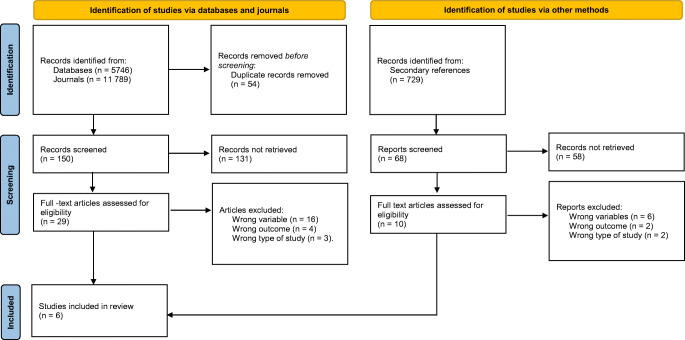
PRISMA flow diagram. Figure 1 shows the PRISMA Flow Diagram illustrating the literature search, identification, screening, assessment for eligibility leading to the final article selection

The performed literature search identified 19 164 results; among the 237 articles screened, 29 full-text articles were assessed for eligibility. Ultimately six articles were included based on the established inclusion and exclusion criteria (Fig. 1). Of these, two studies focused on NICU patients while four focused on PICU patients. The NICU studies were a prospective case-control study and a retrospective cohort study, totaling 2172 patients [23, 24]. Among the PICU studies, three were prospective cohort studies and one was a retrospective cohort study, totaling 42 368 patients [25–28].

Quality assessment of the papers was performed independently by both authors using the BMJ criteria and Cochrane Collaboration’s risk-of-bias tool. Authors collaborated and discussed to give each article a final ranking of “low,” “moderate,” or “high.” All but two articles were given a final ranking of “high quality” [23, 25, 26, 28]. One NICU-focused article scored “moderate” based on an increased risk of observer bias: the cognitive outcomes were measured by the study group’s research psychiatrist, as opposed to an independent psychiatrist [24]. It was not specified whether or not the research psychiatrist was aware of the participants’ “case” or “control” patient status [25]. One PICU-focused study scored “moderate” due to an increased risk of confounding factors: the cause of admission and diagnosis were not analyzed as possible factors relating to cognitive outcomes [26]. Based on the Oxford Centre for Evidence-Based Medicine [21], the levels of evidence ranged from 1b to 2b. All six studies were deemed of sufficiently good quality and validity for their conclusion to be accepted and were further analyzed (Table 1).

**Table 1:** The quality assessment and evaluation of levels of evidence of included articles. Table 1 illustrates the quality assessment procedure based on five assessment criteria and the final grading

Article: title, study type, and publication date	Assessment criteria				Final grading
	Participant selection process	Outcome measurement and follow-up	Risk of bias	Other comments	
Developmental Trajectories in Children with Prolonged NICUa Stays - A Prospective Cohort Study (2016)	Transparent Selection Process: all ECLS-Bc non-preterm neonates hospitalized to a NICUa were retrospectively included at the moment of admission. Inclusion/Exclusion Criteria: clearly defined.	Valid methods of measurement were used: BSIDd, PPVTe, PreCOTPPPf, TEMAg and derived tests from the ECLS-Kh Follow up took place at 9-, 24-months, preschool and kindergarten. Follow up rate was complete.	High measurement precision with significant p-values<0.0001. Confounding and prognostic factors: all relevant factors were analyzed. Selection bias: no risk. Information bias: no risk. Observer bias: no risk.	Indirectness: no risk. Inconsistency: no risk. No intervention or indirect comparison were performed. A dose-response gradient existed.	Final Ranking: High OCEBMi Level of Evidence: 2b JAMA Pediatrics Quality Rating: 3
Factors Associated with Developments Progress of Full Term Neonates who Required Intensive Care - A Prospective Case-Control Study (1989)	Transparent Selection Process: Case patients: all consecutive non-preterm neonates hospitalized to a participating NICUa between 1983-1984 were prospectively included at the moment of admission Control patients: healthy newborns matched based patient characteristics. Inclusion/Exclusion Criteria: clearly defined.	Valid methods of measurement were used: GMDSj were assessed by the study group’s research psychiatrist. Follow up took place at 6 weeks, 6 months and 1 year post-natal. Follow up rate was incomplete but accounted for, constituting a drop out rate of 2.8%.	High measurement precision with significant p-values<0.05. Confounding and prognostic factors: all relevant factors were analyzed. Selection bias: no risk. Information bias: no risk. Observer bias: risk present.	Indirectness: no risk. Inconsistency: no risk. No intervention or indirect comparison were performed.	Final Ranking: Moderate OCEBMi Level of Evidence: 1b JAMA Pediatrics Quality Rating: 2
Risk Factors for Acquiring Functional and Cognitive Disabilities during Admission to a PICUb - A Retrospective Cohort Study (2014)	Transparent Selection Process: all consecutive patients admitted to a participating PICUb between 2009-2010 were retrospectively included at the moment of admission. Inclusion/Exclusion Criteria: clearly defined.	Valid methods of measurement were used: PCPCk and POPCl were measured by PICUb personnel at admission and at discharge. Follow up only took place at discharge. Follow up rate was complete.	High measurement precision with significant p-values<0.01. Confounding and prognostic factors: all relevant factors were analyzed. Selection bias: no risk Information bias: no risk Observer bias: minimal risk.	Indirectness: no risk. Inconsistency: no risk. No intervention or indirect comparison were performed. A dose-response gradient existed.	Final Ranking: High OCEBMi Level of Evidence: 2b JAMA Pediatrics Quality Rating: 3
The Impact of Admission to a PICUb assessed by Means of Global and Cognitive Performance Scale - A Prospective Cohort Study (2007)	Transparent Selection Process: all consecutive patients admitted to the HCPAm PICUb between 2002-2005 were prospectively included at the moment of admission. Inclusion/Exclusion Criteria: clearly defined.	Valid methods of measurement were used: PCPCk and POPCl were measured by one of the study’s authors at admission and discharge. Interobserver agreement was assessed during a pilot study and returned a high level of reliability. Follow up took place at discharge. Follow up rate was incomplete: the drop out rate was 6.3% and caused by death.	High measurement precision with significant p-values<0.05. Confounding and prognostic factors: most relevant factors were analyzed with the exception of cause of admission and diagnosis. Selection bias: no risk. Information bias: no risk. Observer bias: no risk.	Indirectness: no risk. Inconsistency: no risk No intervention or indirect comparison were performed. A dose-response gradient existed.	Final Ranking: Moderate OCEBMi Level of Evidence: 1b JAMA Pediatrics Quality Rating: 2
Relationship of Illness Severity and Length of Stay to Functional Outcomes in the PICUb - A Multi-Institutional Prospective Cohort Study (2000)	Transparent Selection Process: all consecutive patients admitted to participating PICUsb in 1999 were prospectively included at the moment of admission. Inclusion/Exclusion Criteria: clearly defined	Valid methods of measurement were used: PCPCk and POPCl were measured by PICU personnel at admission and discharge. Interobserver agreement was tested twice, 6 months apart, and returned a high level of reliability. Follow up rate was incomplete: the drop out rate was 4.6% and caused by death.	High measurement precision with significant p-values<0.05. Confounding and prognostic factors: all relevant factors were analyzed. Selection bias: no risk: Information bias: no risk Observer bias: no risk	Indirectness: no risk. Inconsistency: no risk. No intervention or indirect comparison were performed. A dose-response gradient existed.	Final Ranking: High OCEBMi Level of Evidence: 1b JAMA Pediatrics Quality Rating: 2
Assessing the Outcome of Pediatric Intensive Care - A Prospective Cohort Study (1992)	Transparent Selection Process: all consecutive patients admitted to the Arkansas Children’s Hospital PICUb between 1989-1990 were prospectively included at the moment of admission. Inclusion/Exclusion Criteria: clearly defined.	Valid methods of measurement were used: PCPCk and POPCl were measured by the study’s nurse at admission and discharge. Interobserver agreement was tested and returned a high level of reliability. Follow up rate was incomplete: the drop out rate was 5.8% and caused by death.	High measurement precision with significant p-values<0.05. Confounding and prognostic factors: all relevant factors were analyzed. Selection bias: no risk: Information bias: no risk Observer bias: no risk	Indirectness: no risk. Inconsistency: no risk. No intervention or indirect comparison were performed. A dose-response gradient existed.	Final Ranking: High OCEBMi Level of Evidence: 1b JAMA Pediatrics Quality Rating: 2

^a^NICU: neonatal intensive care unit

^b^PICU: pediatric intensive care unit

^c^ECLS-B: early childhood longitudinal study-birth

^d^BSID: Bayley Scales of Infant Development

^e^PPVT: Peabody Picture Vocabulary Test

^f^PreCOTPPP: preschool comprehensive test of phonological and print processing

^g^TEMA: test of early mathematical ability

^h^ECLS-K: early childhood longitudinal study-kindergarten

^i^OCEBM: Oxford Centre of Evidence Based Medicine

^j^GMDS: Griffiths Mental Development Scales

^k^PCPC: pediatric cerebral performance category scale

^l^POPC: pediatric overall performance category scale

^m^HCPA: Hospital Acadêmico de Porto Alegre

### cognition in the NICU population

Two articles were identified and included which examined NICU hospitalization in relation to cognitive developmental outcomes in NICU survivors. The first study measured developmental outcomes using the Bayley Short Form Research Edition Tests at 9-, 24-months, preschool, and kindergarten [23]. A significant relationship was found at 9- (*p*<0.001) and 24-months (*p*<0.01), with significant differences in adjusted mean Baley Test scores between patients with shorter or longer stays (*p*<0.001) [23]. Each additional week in the NICU increased the odds of scoring in the lowest 10th percentile at 9- (OR 1.08, 95% CI 1.034–1.122) and at 24-months (OR 1.11, 95% CI. 1.065–1.165) [23]. This relationship was lost at preschool and kindergarten follow-up moments [23].

The second study measured developmental outcomes using the Griffiths Child Development Scales at 6-weeks, 6- and 12-months [24]. Significant differences between case and control patients were only found at 12-months in five of the seven elements of Griffith’s Scales: general quotient (*p*<0.03), mental age (*p*<0.03), hearing/speech (*p*<0.04), hand-eye coordination (*p*<0.005), and motor development (*p*<0.04) [24]. At 1 year, 35% of the variance of general quotient scores was explained by the length of hospital stay (*p*<0.0001) [24]. Repeated hospital admission, number of days on a ventilator, postnatal complications, socioeconomic status, and maternal mental health had a negative relationship with cognitive outcomes [23, 24].

Neither study found a relation with Apgar scores, gestational categories or race [23, 24]. These studies both conclude that NICU hospitalization not only has a negative relationship but also a dose-gradient effect, up until 24-months post-discharge on various cognitive outcomes (Table 2).

**Table 2:** Summary and review of the results of included articles. Table 2 provides a summary for the objectives, outcomes, methodology and results for all included articles

Article: title, study type and publication date	Objectives and outcomes	Methodology	Results
Developmental Trajectories in Children with Prolonged NICU^a^ Stays - A Prospective Cohort Study (2016)	Objectives: assessing if the length of NICU^a^ admission is a good marker for developmental outcome in NICU^a^ survivors. Evaluation of the role of factors: days on ventilator, Apgar score and SES^c^ factors. Outcomes: cognitive and motor performance.	Participants: 2100 NICU patients who were part of the ECLS-B^d^ dataset. Outcome Measurement: BSID^e^, PPVT, PreCOTPPP, TEMA and derived tests from the ECLS-K^i^ measured at 9-, 24-months, preschool and kindergarten. Main Covariates: gestation category, race, sex, Apgar score, days on ventilator and SES^c^ Statistical Analysis: linear and logistic regression, adjusted mean scores	Significant negative relationship between length of NICU^a^ stay and BSID^e^ at 9- and 24-months (p<0.001) Odds of scoring in the lowest 10th percentile of BSID^e^ at 9- and 24-months increased by 1.08 (95% CI 1.034-1.122) and 1.11 (95% CI 1.065-1.165) respectively. Days on ventilator was the only significant confounding factor.
Factors Associated with Developments Progress of Full Term Neonates who Required Intensive Care - A Prospective Case-Control Study (1989)	Objectives: describing the factors influencing the developmental progress of full-term neonates admitted to the NICU^a^, particularly the role of hospitalization and of maternal separation. Outcomes: intellectual, social and emotional performance.	Participants: Case patients: 43 infants admitted to the NICU between 1983 and 1984 at the Hospital for Sick Children or Queen Charlotte’s Maternity Hospital Control patients: 29 healthy infants matched for baseline characteristics Outcome Measurement: GMDS^k^ measured at 6- and 12-months post-discharge. Main Covariates: birth weight, gestational age, severity of medical condition, days on ventilator, marital relationship, maternal mental health Statistical Analysis: multiple and multivariate regression	Significant negative relationship between length of NICU^a^ stay and five of seven GMDS^k^ elements: general quotient (p<0.03), mental age (p<0.03), hearing/speech (p<0.04), hand-eye coordination (p<0.005) and motor development (p<0.04). 35% of variance between general quotient scores is explained by length of hospital stay (p<0.0001). Maternal mental health was the only significant confounding factor.
Risk Factors for Acquiring Functional and Cognitive Disabilities during Admission to a PICU^b^ - A Retrospective Cohort Study (2014)	Objectives: describing the factors associated with acquired cognitive or global functional impairments in PICU^b^ survivors and identifying a combined set of factors to be used in a clinical setting to identify high-risk PICU^b^ patients for acquiring such impairments. Outcomes: cognitive and global performance.	Participants: 29352 PICU^b^ patients part of the VPS admitted between 2009-2010 Outcome Measurement: PCPC^m^ and POPC^n^ scales measured at admission and discharge. Main Covariates: age, sex, unscheduled PICU^b^ admission, PIM^o^, ventilator use, invasive interventions, trauma diagnosis Statistical Analysis: univariate analysis, multiple logistic regression analysis	Significant negative relationship between PICU^b^ stay and cognitive and global development outcomes (p<0.01). Unscheduled admission (OR 1.52, 95% CI 1.16-2.00), mechanical ventilation (OR 2.83, 95% CI 2.36-3.39), renal replacement therapy (OR 2.83, 95% CI 1.73-3.42), and highest risk of mortality category (OR 2.70, 95% CI 2.15-3.40) were independently associated with acquiring cognitive functioning.
The Impact of Admission to a PICU^b^ assessed by Means of Global and Cognitive Performance Scale - A Prospective Cohort Study (2007)	Objectives: assessing the impact of admission to, length of stay on the PICU^b^ and mortality risk on survivors’ cognitive and global development Outcomes: cognitive and global performance.	Participants: 443 patients admitted at the HCPA^p^ PICU^b^ between 2002-2005 Outcome Measurement: PCPC^m^ and POPC^n^ scales measured at admission and discharge. Main Covariates: age, sex, diagnosis, PIM^o^ Statistical Analysis: standard distribution assessments, Kruskal Wallis test	Significant difference was found between discharge delta scores of PCPC^m^ and POPC^n^ outcome scores and PICU^b^ length of stay (p<0.001) and risk of mortality (p<0.001)
Relationship of Illness Severity and Length of Stay to Functional Outcomes in the PICU^b^ - A Multi-Institutional Prospective Cohort Study (2000)	Objectives: assessing the relationships between illness severity, length of PICU^b^ stay and on survivors’ cognitive and global development survivors. Outcomes: cognitive and global performance.	Participants: all admissions within 12 consecutive months to 16 participating PICUs^b^ of the PCC^q^ Study Group, totalling 11104 patients Outcome Measurement: PCPC^m^ and POPC^n^ scales measured at admission and discharge. Main Covariates: age, PRISM^r^, operative status, trauma status, hospital type Statistical Analysis: Kruskal Wallis test, multivariate regression analysis	Significant difference was found between discharge and delta PCPC^m^ and POPC^n^ outcome scores and PICU^b^ length of stay (p<0.01) and risk of mortality (p<0.01). Increased length of stay was associated with worsened PCPC^m^ and POPC^n^ outcome scores.
Assessing the Outcome of Pediatric Intensive Care - A Prospective Cohort Study (1992)	Objectives: assessing the association between risk of mortality, morbidity, length of PICU^b^ stay and cognitive and development in survivors. Outcomes: cognitive and global performance.	Participants: all admissions to Arkansas Children’s Hospital PICU^b^ between 1989-1990, totalling 1469 patients Outcome Measurement: PCPC^m^ and POPC^n^ scales measured at admission and discharge. Main Covariates: age, PRISM, discharge care needs, injury status Statistical Analysis: one-way analysis variance procedure, Student-Newman-Keuls multiple comparison test	Significant difference was found between discharge and delta PCPC^m^ and POPC^n^ outcome scores and PICU length of stay (p<0.0001), greater discharge care needs (p<0.0001), and greater PRISM scores (p<0.0001).

^a^NICU: neonatal intensive care unit

^b^PICU: pediatric intensive care unit

^c^SES: socioeconomic factors

^d^ECLS-B: early childhood longitudinal study-birth

^e^BSID: Bayley Scales of Infant Development

^f^PPVT: Peabody Picture Vocabulary Test

^g^PreCOTPPP: preschool comprehensive test of phonological and print processing

^h^TEMA: test of early mathematical ability

^i^ECLS-K: early childhood longitudinal study-kindergarten

^j^OCEBM: Oxford Centre of Evidence Based Medicine

^k^GMDS: Griffiths Mental Development Scales

^l^VPS: Virtual PICU Performance System data network

^m^PCPC: pediatric cerebral performance category scale

^n^POPC: pediatric overall performance category scale

^o^PIM: Pediatric Index of Mortality

^p^HCPA: Hospital Acadêmico de Porto Alegre

^q^PCC Study Group: Pediatric Critical Care Study Group of the Society of Critical Care Medicine

^r^PRISM: pediatric risk of mortality

### cognition in the PICU population

Four articles were identified and included which examined PICU hospitalization in relation to PICU survivors’ cognitive and general functioning. Cognitive and global functioning outcomes were measured using the PCPC and POPC Scales respectively at admission and at discharge [25–28]. All four studies found a significant relationship between the length of PICU hospitalization and cognitive and global outcomes [25–28]. Low discharge PCPC scores were associated with length of stay (*p*<0.01) and risk of mortality (*p*<0.0001) [25–28]. Analysis of delta scores between admission and discharge PCPC scores showed that greater delta scores were associated with length of stay (*p*<0.001) and risk of mortality (*p*<0.001) [26, 28].

Additionally, factors were identified characterizing patients at a higher risk for worsened cognitive outcomes at discharge: PICU stay >2.5 days, unscheduled PICU admission and invasive mechanical ventilation [25]. Amongst patients with worsened cognitive or global outcomes 49.2% and 29.9% respectively had all three risk factors (*p*<0.01) [25]. The prevalence of acquired cognitive impairment in patients with all three risk factors was 23.0% (*p*<0.01) [25]. Other factors were shown to have a negative relationship with cognitive ability were increased mortality risk, invasive interventions, and trauma, neurological or oncological diagnosis [25–28].

These results show the existence of a relationship between PICU hospitalization and cognitive outcomes at discharge with the added component of a dose-gradient effect and allow for the identification of patients at the greatest risk for acquired cognitive impairment (Table 2).

## Discussion

This systematic review examines the association between cognition and cognitive development in NICU and PICU survivors, and ICU hospitalization. Previous research had established that ICU hospitalization and pediatric critical illness has a negative impact on mental health, which can subsequently impact cognitive functions. However, the effect of hospitalization in and of itself on cognitive status and development had not been considered, thereby ignoring possible causes of cognitive impairment and possible points of improvement.

The results have established that a negative association exists between NICU and PICU hospitalization, and the cognitive status and development outcomes of survivors, both in the short and in the long term. A dose-response effect was found, while repeated hospitalization, unscheduled hospitalization, and invasive or painful interventions were identified as the elements which most contribute to this negative association.

Several limitations were identified and their significance for the results of this systematic review was examined. It is necessary to note that there is a general lack of research concerning this topic: despite an extensive literature search a mere six articles were found and even these six articles do not examine the topic in its full extent. The quality of the studies was not considered a limitation: the quality assessment that all six were of sufficiently good quality and validity for their conclusions to be accepted.

The first limitation was the length of follow-up: to allow for both the identification and observation of the evolution of the association between cognition and hospitalization, it was important to evaluate cognitive outcomes following admission and in the period afterward. Although the PICU-focused studies all identified a negative association between hospitalization and cognition at discharge, none had follow-up moments extending past discharge. This limitation renders it difficult to evaluate the long-term effects of hospitalization and the progression thereof on cognition. However, the NICU-focused studies both identified a similar association and found that the cognitive impairments lasted up until 2 years post-discharge.

The second limitation was the lack of research regarding which aspects of hospitalization, in particular, were responsible for the negative association with cognition. These studies aimed to detect the existence of an association and therefore only made a restrained effort to understand which elements of hospitalization were most important. Important negative stimuli were identified, namely unscheduled admission, repeated admission, and invasive mechanical ventilation or interventions. The absence of positive stimuli, such as the presence of family or exposure to age-appropriate educational activities, was not examined and remains hypothesized. These studies have succeeded in detecting the negative association between hospitalization and cognition; however, it is necessary to acquire a better understanding of which hypothesized features of hospitalization are relevant in order to properly address this problem.

These limitations are considered productive: while ignorance regarding a non-existent problem is irrelevant, this research and these limitations show that there is insufficient information regarding a very real and important problem which must be further explored.

Two main approaches should be adopted to limit cognitive impairments and, ideally, promote cognition. The first is prevention, by improving ICU hospitalization and addressing patient and hospital-related risk factors for cognitive impairment. The second is management, by appropriate follow up and treatment of survivors with or at risk of cognitive impairment post-discharge. Future research will have to focus on these aspects, however existing research could be used as a stepping stone.

Improving hospitalization first requires future research to pinpoint which aspects have the most impact on cognitive status and development. Based on this systematic review, the most important risk factors are unscheduled or repeated hospital admission, and invasive or painful interventions. Further research has shown that certain interventions common during PICU hospitalizations are linked with cognitive impairment, including ECMO treatment, mechanical ventilation, repeated surgeries or use of sedation and pain medications [29–32]. Psychological stress has also been found to contribute to cognitive impairments, with important sources also including separation from the family and familiar environment, reducing sources of fear and improving communication with patients and families [33]. Sources of fear typically revolve around pain and the unknown and are improved through appropriate explanations and communications [339]. Studies interviewing patients and families have found that uniformed personnel and hospital design contribute to stress [17]. Reducing the PICU-related interventions and improving family, access to social media, and appropriate, transparent interactions are the best ways to address these factors [34–36]. Patients, regardless of age and sex, also have a preference for a blue color scheme and thematic design focused around comfort and a “home away from home feel” [17, 18].

In addition to reducing negative factors relating to hospitalization, improving positive cognitive stimuli is important. A systematic review by Hussey et al found that interventions on the NICU focusing on sensory stimulation had a significantly positive impact: sensory skills, sleep, behavior, communication and organization skills were all improved in the short and long term [37]. Older children benefit from access to exercise, play and education however these are a challenge to implement in the context of illness and hospitalization.

It is interesting to note that the risk factors for cognitive impairment identified here parallel risk factors for pediatric delirium, particularly hospitalization duration, invasive interventions, use of pain medication and sedation, foreign environment and a lack of familial presence. [38–40] As such, delirium-prevention bundles could be effective in reducing cognitive impairment. [40–42]

To conclude, this systematic review established that ICU hospitalization in and of itself has a significant negative impact on the cognitive status and development of survivors, both in the short and long term. The results have significant consequences for future research and clinical practice. Future research must evaluate which aspects of hospitalization are most relevant, on the ability to extend the findings to non-ICU hospitalization, and on possible measures to mitigate the impact of hospitalization on cognition.

In clinical practice, professionals need to be increasingly aware of the short- and long-term cognitive impairments which may follow hospitalization. Based on the findings of this systematic review, it is recommended that NICU and PICU patients be screened to identify those at risk for cognitive impairments or delays post-discharge. Results suggest patients with an ICU hospitalization length of over 2.5 days, and two or more of the following factors—increased mortality risk, invasive interventions, neurological diagnosis, oncological diagnosis, postnatal complications or decreased maternal mental health status—should be considered as high risk. These patients should be appropriately followed up and treated as necessary post-discharge. These recommendations, accompanied by further research, will be an important step towards guaranteeing cognition, learning ability and quality of life of NICU and PICU survivors.

## Data Availability

All data used for this systematic review is available, ensuring complete data transparency.

## Abbreviations

ICU: intensive care unit
NICU: neonatal intensive care unit
PCPC: pediatric cerebral performance category scale
PICU: pediatric intensive care unit
POPC: pediatric overall performance category scale
